# A Transporter Interactome Is Essential for the Acquisition of Antimicrobial Resistance to Antibiotics

**DOI:** 10.1371/journal.pone.0152917

**Published:** 2016-04-06

**Authors:** Yonatan Shuster, Sonia Steiner-Mordoch, Noemie Alon Cudkowicz, Shimon Schuldiner

**Affiliations:** Department of Biological Chemistry, Alexander Silberman Institute of Life Sciences, Hebrew University, Jerusalem, Israel; Institut National de la Recherche Agronomique, FRANCE

## Abstract

Awareness of the problem of antimicrobial resistance (AMR) has escalated and drug-resistant infections are named among the most urgent problems facing clinicians today. Our experiments here identify a transporter interactome and portray its essential function in acquisition of antimicrobial resistance. By exposing *E*. *coli* cells to consecutive increasing concentrations of the fluoroquinolone norfloxacin we generated in the laboratory highly resistant strains that carry multiple mutations, most of them identical to those identified in clinical isolates. With this experimental paradigm, we show that the MDTs function in a coordinated mode to provide an essential first-line defense mechanism, preventing the drug reaching lethal concentrations, until a number of stable efficient alterations occur that allow survival. Single-component efflux transporters remove the toxic compounds from the cytoplasm to the periplasmic space where TolC-dependent transporters expel them from the cell. We postulate a close interaction between the two types of transporters to prevent rapid leak of the hydrophobic substrates back into the cell. The findings change the prevalent concept that in Gram-negative bacteria a single multidrug transporter, AcrAB-TolC type, is responsible for the resistance. The concept of a functional interactome, the process of identification of its members, the elucidation of the nature of the interactions and its role in cell physiology will change the existing paradigms in the field. We anticipate that our work will have an impact on the present strategy searching for inhibitors of AcrAB-TolC as adjuvants of existing antibiotics and provide novel targets for this urgent undertaking.

## Importance

Antibiotic resistance is a growing global public health concern and was recently defined as among the most urgent problem facing physicians today. To achieve high-level resistance, multiple mutations accumulate sequentially, each providing a small but distinct increase in fitness. Here we identified transporters that are essential for the acquisition of resistance to quinolones and chloramphenicol by decreasing the cytoplasm concentration to values that allow fixation of single mutations. To achieve this different types of transporters must work in a concerted mode, some of them supplying the antibiotic to the periplasm where others remove it to the cell exterior. The interaction is essential for acquisition of high-level, clinically significant antibiotic resistance.

## Introduction

Multidrug efflux transporters (MDTs) are a ubiquitous class of integral membrane proteins of vast clinical interest because of their strong association with human disease and pharmacology [1, 2]. In a model organism such as *Escherichia coli* more than 20 different MDTs have been shown to confer resistance to one or more drugs [3]. MDTs have been proposed to play a role in the emergence of antimicrobial resistance (AMR).

Awareness of the problem of AMR has become acute in both academic [4, 5] and public circles and threat reports have been issued naming drug-resistant infections among the most urgent problems facing clinicians today [6]. The suggested role of MDTs is based on the ability of many to confer medium level resistance when overexpressed and increased susceptibility when inactivated [3, 7, 8]. Moreover, global responses to stress upon exposure to antibiotics bring about an increase in the expression of multiple MDTs [9–11]. Bacteria that overexpress MDTs, including Enterobacteriaceae, *P*. *aeruginosa* and *S*. *aureus*, have been isolated in the clinics [9]. Furthermore, a large number of studies using inhibitors of MDTs and genetic tools support the contention that MDTs provide a central survival strategy for the bacterial organism and they are involved in AMR. Thus, for example, inhibition of the multidrug transporter NorA prevents emergence of Norfloxacin resistance in *Staphylococcus aureus* [12]. Additionally, inactivation of major efflux systems lowers the frequency of emergence of fluoroquinolone resistant mutants and strains carrying specific target- site mutations are no longer resistant if some MDTs are inactivated [13].

However, clinical isolates usually display higher levels of resistance than those that can be supported by MDTs and, for most antibiotics, multiple mutations are required to develop high levels of resistance [14–17]. Moreover, most of the studies performed thus far in this context are based on exposure to fixed drug doses that permit only the growth of resistant mutants. Naturally, this approach identifies only a single adaptive step and does not reveal how multiple mutations can accumulate sequentially to confer robust resistance.

Our understanding of the interaction of the large MDT cohort and its role in the intrinsic and the acquired resistance to antibiotics is the focus of this work. We demonstrate the need for cooperation between various MDTs to acquire high-level resistance to quinolones and to chloramphenicol. This was done by evaluating the role of the AcrAB-TolC complex and other rationally chosen MDTs in the process of resistance acquisition in bacteria. By exposing *E*. *coli* cells to consecutive increasing concentrations of the fluoroquinolone norfloxacin we simulated in the laboratory a possible path of evolution that leads to high-level AMR. This approach allowed generation of highly resistant strains that carried multiple mutations that accumulate sequentially to confer strong resistance~600 fold higher than the naïve strains. Whole genome sequencing of the evolved strain revealed that most of the mutations accumulated were mutations identified also in clinical isolates. Typical experiments lasted for about 18–20 days until the concentration of the drug could no longer be increased because of solubility problems. Cells bearing a triple knockout in three single-component transporters known to be involved in quinolone transport develop a much weaker resistance, ~50-fold lower than the wild type. Remarkably, cells bearing a knockout in AcrB develop a level of resistance only slightly lower than that observed in the wild type cells. We provide evidence that, in this strain, expression of other TolC-dependent transporters increases and their activity is sufficient for the development of high-level resistance.

We conclude that the functional interaction of various types of MDTs provides an essential first-line defense mechanism, preventing drugs from reaching lethal concentrations, until a number of stable, more efficient alterations occur, that allow survival in the presence of that agent.

## Materials and Methods

### Strains and plasmids

*E*. *coli* BW25113 strain was used throughout this work. BW25113 Δ*emrE*, Δ*mdfA*, Δ*mdtM* and Δ*acrB*, individual knockouts and combinations were described by Tal and Schuldiner [18] and prepared essentially according to Datsenko and Wanner [19, 20].

### Evolution Process

The *in-vitro* evolution process involved growth in LB medium overnight and then dilution of the culture to 20 mL LB-KP_i_ medium (LB medium containing 70 mM potassium phosphate, pH 7.4) containing 0.05 μM norfloxacin, a sub-lethal concentration. The cells were then grown overnight and cultures that reached at least A_600_ = 1 were further diluted to LB-KP_i_ containing twice the norfloxacin concentration, grown overnight yet again and examined for growth. The process was continued with a daily two-fold increase in the norfloxacin concentration for about 18–20 days till it could no longer be increased because we reached the maximum solubility in water (400 μM). In case the culture did not reach A_600_ = 1 after overnight growth, the cells were allowed to grow one more night. If no growth was recorded, the cells were moved to a previous, lower, concentration of norfloxacin and allowed to grow again. The sequential increase was stopped if the cells reached a concentration in which no growth was recorded after 48 hours.

Throughout the evolution process, all strains were tested daily using PCR to verify the stability of all knockouts. Primers complementary to chromosomal sequences flanking the knockout region were used and the size of the product was compared to that of the wild type strain and the calculated value for each given knockout.

At the end of the experiment chromosomal DNA was prepared using the NucleoSpin^®^ Tissue kit (Macherey&Nagel, Düren, Germany) and the genome was sequenced using 150-bp paired-end reads on the Illumina NextSeq500 platform. Reads were aligned to the *E*.*coli* BW-25113 genome (GenBank accession CP009273.1) and variants were called using the Chipster software [21].

### Determination of resistance levels in liquid medium

For resistance in liquid medium, overnight cultures were diluted into LB medium, grown to early logarithmic phase, and diluted to A_600_ = 0.01 in LB-KP_i_ medium and the indicated concentrations of toxic compounds in a 96-well plate (Nunc) at 37°C with constant shaking. A_600_ readings were taken every hour for 12 h using a Synergy 2 Microplate Reader (BioTek). Plots of bacterial growth vs. drug concentrations were then fitted using OriginLab’s Origin Pro 9.1 software. In the late stages of the experiment levels of resistance were extremely high and we were not able to determine MIC values, therefore, we calculated for all the time points and all the strains the IC_50_ value derived from the fit equation. In the case of the highly resistant cells, this is only an estimate due to the fact that inhibition was only partial.

The evolution experiment was repeated three times with practically identical results. Whole genome sequencing was performed only in the experiment shown in this manuscript. The IC_50_ values shown are from one experiment. For each time point the IC_50_ value was determined at least twice.

### Determination of resistance levels on solid medium

For testing resistance on solid medium, cells were grown overnight at 37°C in LB medium. The cultures were diluted to A_600_ = 0.2, and 5 μL of serial dilutions of the culture was spotted onto LB plates containing 70 mM KP_i_, pH 7.4, with or without the addition of the indicated concentrations of norfloxacin. Growth was visualized after overnight incubation at 37°C. In all cases growth of all the strains in the absence of antibiotics was similar. All experiments were repeated at least twice.

### Determination of transcript levels

The bacterial total RNA was extracted using PureLink^®^ RNA Mini Kit (Ambion) and cDNA was synthesized from 1.0 μg of RNA using the High Capacity RNA-to-cDNA^™^ kit (Applied Biosystems^™^) and stored at -80°C.

The cDNA levels of target genes were determined by relative quantitative real time PCR using the StepOne Plus Real Time PCR system (Applied Biosystems) in a 20 μl reaction for 40 cycles. Reactions were prepared using the Fast SYBR^®^ Green Master Mix the primers shown in S1 Table. Analysis of gene expression was performed using the comparative C_T_ method [22], normalized to the control *gapA* gene.

## Results

### The experimental paradigm for acquisition of resistance

**Fig 1:**
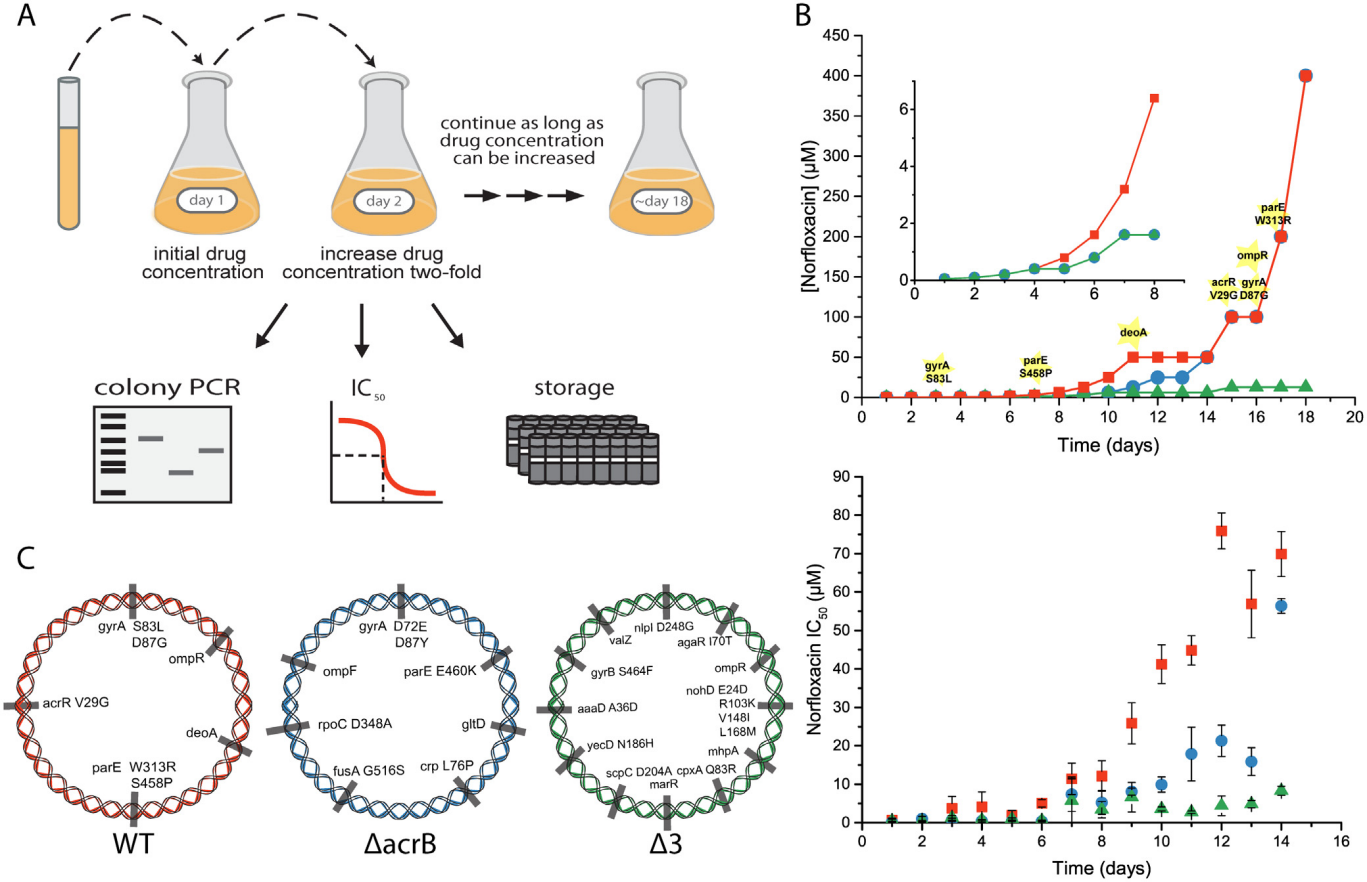
Experimental design for the evolution of high-level resistance to norfloxacin. **A.** Cells were grown overnight in the presence of 0.05 μM norfloxacin and cultures that reached at least A_600_ = 1 were further diluted to LB-KP_i_ containing twice the norfloxacin concentration. The process was continued with a daily two-fold increase in the norfloxacin concentration for 18 days till the concentration (400 μM) could no longer be increased because of solubility problems. Throughout the evolution process, all strains were stored, tested daily using PCR to verify the stability of all knockouts and IC_50_ values were determined. **B top**: Time course of the experiment with wild type (red), Δ*acrB* (blue) and Δ3 (Δ*mdfA*, Δ*mdtM* and Δ*emrE*, green); the mutations appearing during the experiment in the wild type strain are highlighted; **B bottom**: IC_50_ values for each strain determined daily. The IC_50_ values for days 14–18 are not shown because inhibition of growth of WT and Δ*acrB* strains was too low for accurate determination and they are, at least, 400 μM. **C:** Mutations identified in the genome of each one of the strains at the end of the evolution process.

Multistep evolution was carried out in the lab by exposure of cells to sublethal concentrations of norfloxacin that were daily doubled till reaching a concentration that could not longer be increased (Fig 1A).

An example of one of three repeats of the *in-vitro* evolution experiment is shown in Fig 1B-top. The initial concentration of norfloxacin used was 0.05 μM. After overnight growth, cells that reached an A_600_ of 1 or higher were diluted into fresh medium containing a concentration two fold higher. The wild type strain was able to grow even after stepwise increase of 1000 fold the concentration of norfloxacin during the first 11 days of the experiment. At this stage it was not possible to increase further the concentration and the cells were kept at 50 M for 2 days. At day 14 the cells were able to cope again with a two-fold daily increase till it was technically impossible to continue with the experiment because we have reached, at 400 μM, the maximum solubility of norfloxacin in water. The resistance to norfloxacin was quantitated daily by measurement of growth rate at various concentrations and calculation of the IC_50_ value. Resistance increased gradually as expected from a process that is assumed to result from accumulation of multiple mutations (Fig 1B-bottom and see also below). Thus, IC_50_ was 0.69±0.4 μM for the naïve cells and about 70±5 μM after 14 days, i.e. ~100 fold increase. For the 14–18 days evolved strains it was not possible to determine reliable IC_50_ values because inhibition of growth at the limit of solubility in water (400 μM) was lower than 50%. Therefore, we assume IC_50_ values of at least 400 μM that means an increase of ~600 fold or more during the evolution experiment.

The high-level resistance to norfloxacin displayed by the evolved strain (WT-EV18) is shared with other quinolones. We tested nalidixic acid with an IC_50_ value at least 2.5 mM (again, limited by solubility) and ofloxacin, IC_50_ 150 μM (at least 60 and 3000 times higher than the naïve strain, respectively).

Many clinical isolates of *E*. *coli* cells highly resistant to fluoroquinolones have been isolated and the mutations that support such a high-level resistance have been identified [14, 23–26]. It is reasonable to imagine that in clinical settings there are a large variety of pathways to high-level resistance, not necessarily similar to what we have devised here. To test whether our experimental paradigm mimics the properties detected in the clinical strains we sequenced the genome of WT-EV18 (Fig 1C). We identified the classical and well-known mutations in the *gyrA* gene coding for the DNA gyrase, subunit A (S83L and D87G), in the *parE* gene, coding for Topoisomerase IV, subunit B (S458P and W313R), in the *acrR* gene, the transcriptional repressor for acrAB (V29G), in *ompR*, regulator of porin synthesis (insertion-deletion) and in *deoA*, coding for thymidine phosphorylase (synonymous mutation). In addition, two intergenic mutations may affect levels of expression of YghW, a protein with unknown function and PagP, an outer membrane protein part of an operon that has been shown to affect the phenotype of DNA gyrase and topoisomerase mutations [27].

The sequence of the appearance of the mutations provides interesting information (Fig 1B top). One of the classical mutations (S83L) in the DNA gyrase, appears very early in the evolution not only in the experiment shown here but also in three other replicas. This mutation cannot provide by itself the full high-level resistance achieved in our experiments and described in studies of clinical isolates. Previous studies that support the role of MDTs in AMR are based on exposure to fixed drug doses that select for strains carrying the above mutation. There is an excellent correlation between the appearance of new mutations and increase in resistance (Fig 1B top) and the study of the mechanism by which the mutations identified here contribute to resistance should provide powerful tools to combat high-level AMR.

### The role of MDTs in the process of acquisition of resistance

Having established an experimental paradigm that mimics one of the possible pathways in the clinics, we were then able to test the role of MDTs in the process of acquisition of resistance. For this purpose we chose to work with two mutant strains in parallel to the wild type. An *acrB* nil mutation was used because of the well-known major role of the AcrB-TolC type of transporters in Gram-negative bacteria. Since the activity of AcrB-TolC depends on the supply of substrates from the cytoplasm, we also generated a triple knockout of genes coding for suppliers: the *mdfa*, *mdtM* and *emrE* genes were chosen because of the reported involvement of MdfA and MdtM in resistance to quinolones [3, 28]; EmrE was chosen because of the finding that mutants bearing a single mutation conferred robust resistance to quinolones [29]. We tested the triple mutant in detail rather than single ones because of the redundancy in function that may provide backup as previously shown for other toxic compounds [18].

Strikingly, in a series of experiments similar to that described for the wild type, the triple knockout (Δ3) was not able to withstand a continuous increase in the norfloxacin concentration and multiple efforts failed to increase it above 12.5 μM (Fig 1B-top). Notably and unexpectedly, the Δ*acrB* strain displayed an intermediate behavior during the first two weeks but then behaved practically identical to the wild type cells and was capable of overnight growth even in the presence of 400 μM norfloxacin (Fig 1B-top).

For quality control, routine PCR reactions were carried out daily with primers designed to confirm the existence of the knockout mutations. Moreover, the whole genome was sequenced at the end of the 18 days experiment (Fig 1C). As mentioned above for the WT and seen in Fig 1C, also the nil strains developed the resistance gradually because of the need to accumulate multiple mutations (Fig 1B-bottom). Δ*acrB* displayed relatively high levels of resistance, IC_50_ 56±2 μM after two weeks, while the IC_50_ value for the triple knockout increased during the first 7 days but then fluctuated between 5–10 μM even after 18 days. As in the case of the WT, in the last four days it was not possible to determine reliable IC_50_ values for the Δ*acrB* strains because inhibition of growth at the limit of solubility in water (400 μM) was lower than 50%. Therefore, we assume IC_50_ values of at least 400 μM. Growth rates in the absence of norfloxacin were practically identical for all strains and did not change during the evolution experiment.

As in the wild type strain, also in the Δ*acrB* strain the mutations accumulated included the classical targets of quinolones *gyrA* and *parE* (Fig 1C) and *ompF* that may affect the synthesis/function of the OmpF porin. Regarding the mechanism by which all the other mutations confer resistance, we can only speculate, at the moment, from what is known about the genes from the literature. The gene *crp* codes for a c-AMP activated global transcription factor that regulates expression of over 180 genes, one of them the multidrug transporter MdtEF [30]. In addition we identified a mutation in *rpoB* coding for the ß-subunit of the RNA polymerase and *gltD*, coding for the small subunit of the Glutamate synthase, two enzymes previously identified as involved in gene expression under oxidative stress [31, 32] such as the one induced by quinolones. The role of these and the other mutations detected in the high level resistance needs to be established and further analysis should enlighten the multiple survival strategies that allow bacteria to adapt to challenging environments. Thus, for example, the intergenic mutation before the SOS associated gene *dinI* [33] may affect the binding of transcriptional regulators LexA and NagC and the gene *fusA* codes for an elongation factor that may affect expression of proteins relevant to the ability of this mutant to cope with high antibiotic concentrations.

In the Δ3-EV18 strain we identified a very high number of mutations described in Fig 1C. This finding suggests that the norfloxacin-induced stress in these cells was higher than in WT and Δ*acrB* because of the lack of efficient removal of the drug and subsequently the mutability increases. Each one of the mutations may be providing only a modest contribution to the level of resistance and, therefore, a larger number has to accumulate.

### Low-medium level cross-resistance

**Fig 2:**
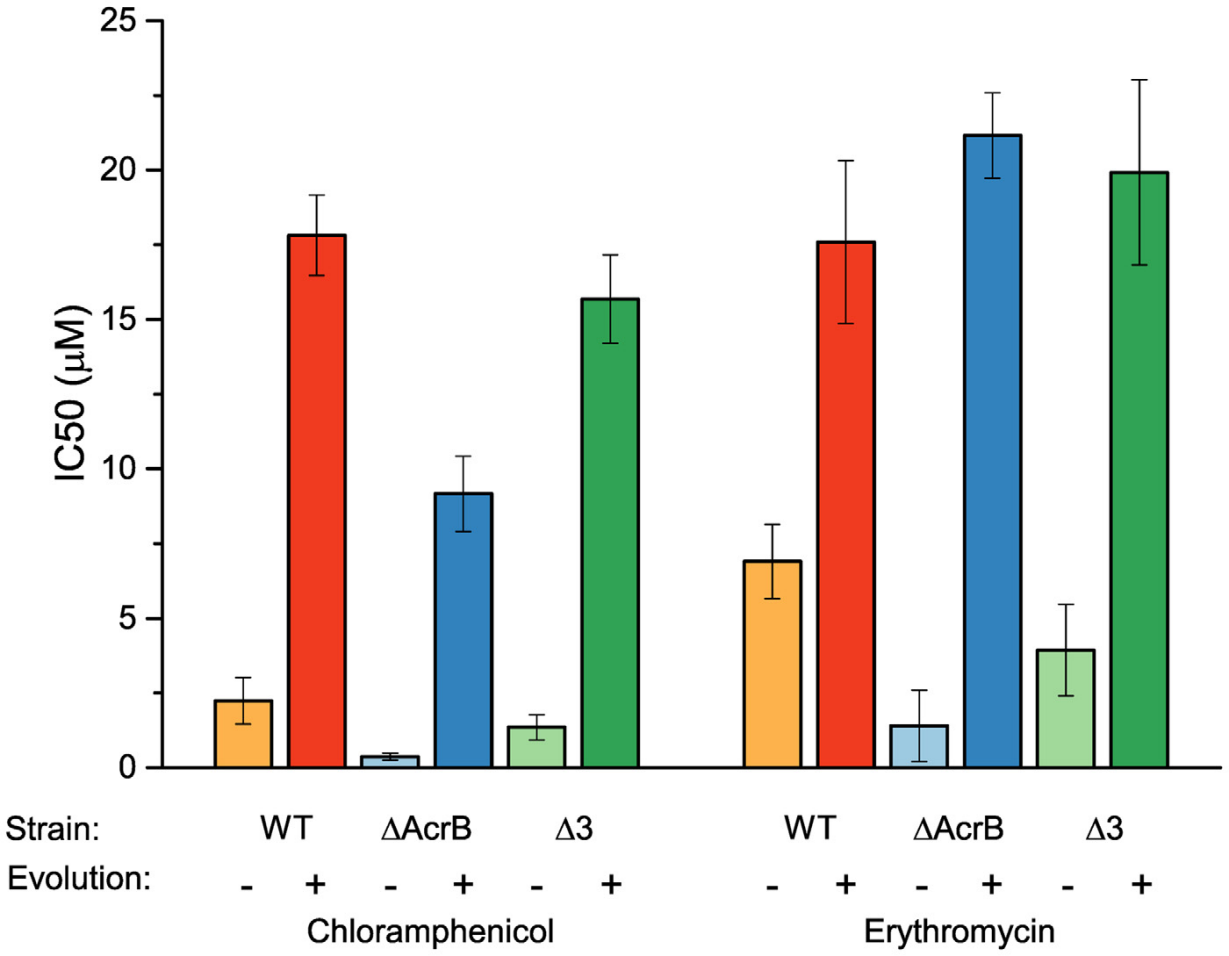
Medium level cross-resistance to antibiotics other than quinolones developed during the evolution experiment. IC_50_ values for erythromycin and chloramphenicol were determined for the naïve and evolved state of each strain.

Exposure to one antibiotic may result in development of cross-resistance to antibiotics other than the ones used during treatment [8]. To test whether the resistance to antibiotics other than quinolones was also affected in our experiment, we examined the susceptibility of the evolved strains to erythromycin (Ery) and chloramphenicol (CM), as representatives of distinctly different antibiotic groups. These experiments are documented in Fig 2 where we observe a significant but relatively modest 5–6 fold increase in the resistance of the WT-EV18 to CM and about 2 fold in the resistance to Ery. Interestingly, cross-resistance was observed also in the Δ*acrB*-EV18 and the Δ3-EV18, where Ery resistance increased 15 fold and 6–7 fold respectively and CM resistance increased 25 fold and 10 fold respectively.

This level of cross resistance is lower than that obtained when chloramphenicol is used in similar experiments as the selecting agent and is most likely due to increased expression of MDTs (see also below). Thus, when evolution is performed by exposure to increasing concentrations of chloramphenicol, wild type and Δ*acrB* cells can grow after 20 days at 400 and 800 μM, respectively (S2 Fig, IC_50_’s 135 and 320 μM, respectively). Also in this case, while AcrB is not required for development of high level resistance, the triple mutant, where genes coding for two proteins known to transport chloramphenicol have been deleted, acquires only low level resistance and cannot grow at concentrations above 50 μM.

### AcrB, an indispensable protein may be dispensed under some conditions

**Fig 3:**
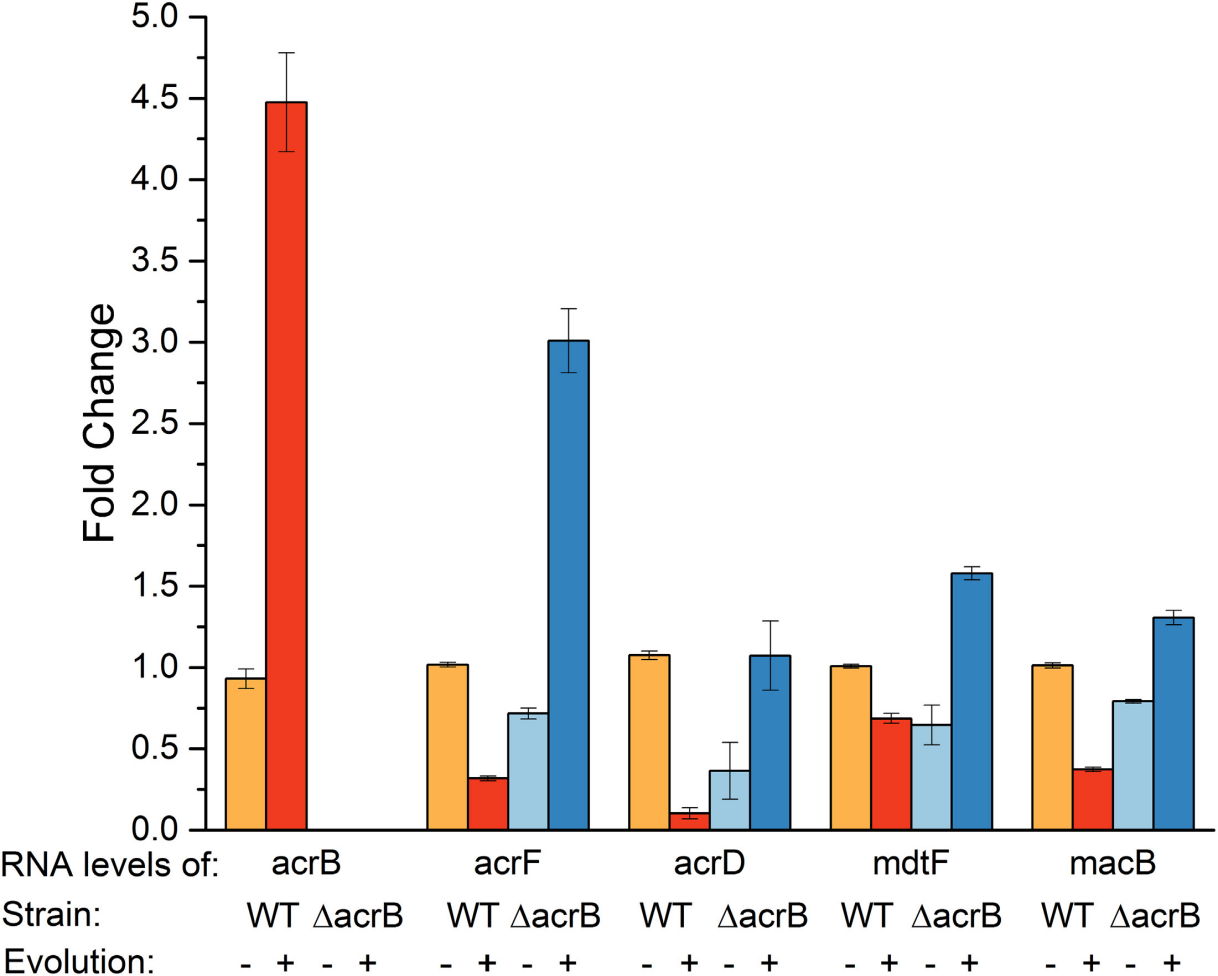
Expression of TolC dependent transporters increases during evolution in the Δ*acrB* strain. The levels of RNA transcripts of the genes *acrB*, *acrD acrF*, *mdtF* and *macB* were determined in WT-EV18 and Δ*acrB*-EV18.

The results shown above demonstrate the central role of the transporters tested in the process of acquisition of high-level resistance to norfloxacin and other quinolones. One of the striking and surprising results of the experiments described above is the finding that the Δ*acrB* strain becomes almost and at least as resistant to norfloxacin and chloramphenicol, respectively, as the WT. Since the central role of the AcrAB-TolC complex is unquestionable and has been documented in many ways, we hypothesized that under extreme conditions its function may be fulfilled by other transporters whose expression is increased under such special conditions. To assess levels of expression, we tested transcript levels of several selected genes encoding for transporters that were previously shown to be responsible for resistance to norfloxacin [3]. We performed qRT-PCR using primers for *acrF*, *acrD*, *macB* and *mdtF*. As expected from reports in the literature, the level of expression of AcrB, as judged from levels of specific RNA detected, in WT-EV18 is about 5 times higher than in the corresponding naïve strain (Fig 3). As anticipated, no RNA coding for AcrB is detected in the nil *acrB* mutant. On the other hand, while expression of AcrF, AcrD, MacB and MdtF is practically the same or somewhat lower in WT-EV18, it is higher, in the Δ*acrB*-EV18 compared to its corresponding naïve strain (~4, 3 and 1.5 fold, respectively) and even higher compared to WT-EV18. We suggest that the increased expression levels of other TolC-dependent MDTs provides sufficient backup to the Δ*acrB* strain and it might explain the high-level resistance achieved by Δ*acrB* -EV18.

### Are the MDTs necessary also for maintenance of the high-level resistance?

#### The suppliers

**Fig 4:**
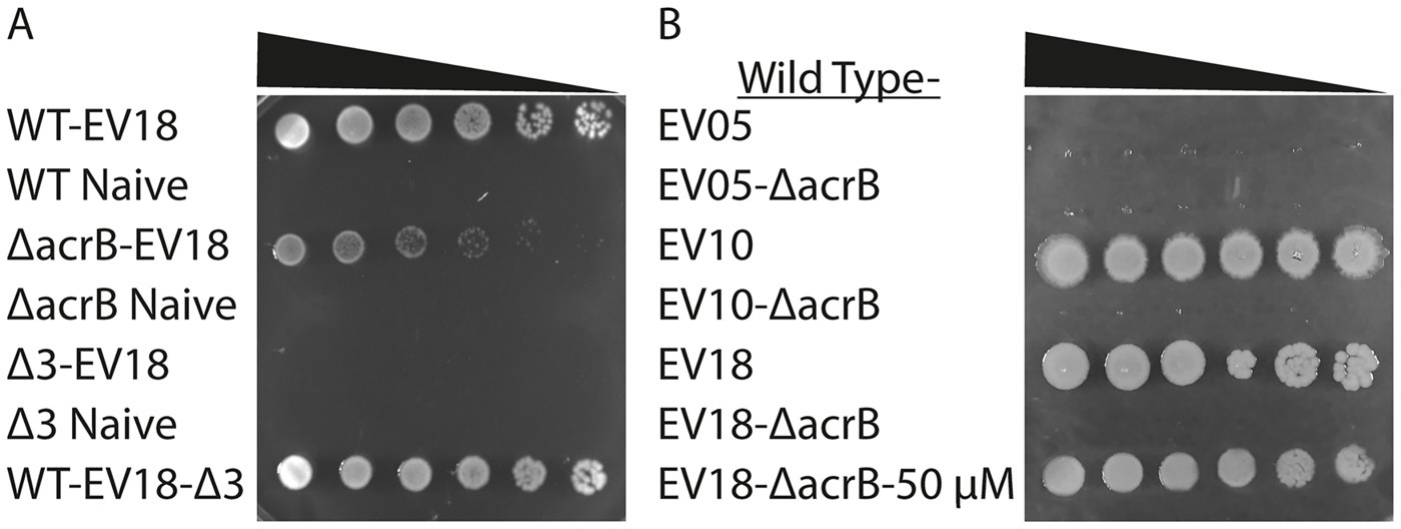
The role of the transporters in maintenance of high-level resistance. A. Solid phenotype in the presence of 400 μM norfloxacin reveals a small but distinct difference in the resistance of Δ*acrB*-EV18 compared to WT-EV18. The suppliers are not required for maintenance of the high-level resistance of WT-EV18 to norfloxacin. B. Solid phenotype in the presence of 30 μM norfloxacin. Nil strains of *acrB* generated after 5, 10 and 18 days of evolution revealed that it plays an important role in maintenance of high-level resistance at every stage. However, after a short exposure to high norfloxacin (50 μM), resistance increases again. Nil strains were generated by deletion of *acrB* (WT-EV18-Δ*acrB*) and of the three suppliers (WT-EV18-Δ3) as described under Experimental Procedures.

The high-level resistance to quinolones in the clinics and in the experiments described here is achieved thanks to the accumulation of multiple mutations, some of them in the direct target of the antibiotic, some in mutations that decrease entrance through the outer wall and yet others in regulatory genes that affect expression of multiple genes. We showed here that the suppliers are required in the process of the acquisition to allow the organism to fix the specific mutation among the many that were generated by the stress situation. To test whether they are required after the high-level resistance is achieved, we generated the same triple nil mutations in the strain that has undergone the evolution (WT-EV18-Δ3) and is capable of growth on 400 μM of norfloxacin. Notably, we could not detect any decrease in resistance of the WT-EV18-Δ3 strain relative to the evolved wt strain because even at 400 μM norfloxacin inhibition was only partial (Fig 4A). Because the partial inhibition observed in this assay does not allow differentiation at the high end, we compared the susceptibility of WT-EV18 and WT-EV18-Δ3 also in a qualitative assay on solid plates. In these experiments 5 μl of serial dilutions of the various strains were spotted on LB-KPi plates containing 400 μM norfloxacin and allowed to grow overnight (Fig 4A). While none of the naïve strains can grow under these conditions, robust growth is observed at all the dilutions of WT-EV18 and WT-EV18-Δ3. This finding clearly demonstrates that once the high-level resistance is achieved none of the selected suppliers play any significant contributing role.

We used this assay also to show again, now in a more graphic way, that, as expected, also growth of Δ3-EV18 was not detectable. Moreover, we can also show that in the case of Δ*acrB* -EV18 growth was not as robust as WT-EV18 indicating a small but distinct difference in susceptibility that was not previously detectable in the liquid growth assay because of the inability to increase the concentration of norfloxacin above 400 μM.

To test whether the medium-level cross-resistance observed can be attributed to specific transporters, we also tested the resistance of the fully evolved strain with the engineered triple nil mutation to erythromycin and chloramphenicol (S1 Fig). In this case, we observed a significant decrease in resistance between the WT-EV18 and its isogenic triple nil strain WT-EV18-Δ3, implying a specific role of the tested transporters in the increased resistance to erythromycin and chloramphenicol.

#### AcrAB-TolC

A different picture emerged when we tested whether the function of AcrAB-TolC complex is needed for the maintenance of the high-level resistance. We generated a nil *acrB* mutation in WT-EV18 (WT-EV18- Δ*acrB*) and we found that the resistance of this strain, albeit still ~10 fold higher than the naïve strain, decreased significantly by at least one order of magnitude (Fig 4B). Remarkably, however, the WT-EV18-Δ*acrB* strain was immediately able to grow at 25 μM norfloxacin and withstood a further increase in the concentration to 50 μM. After additional 48 h of growth at the latter concentration the IC_50_ of these cells increased more than 4-fold to 35.8±11 μM.

## Discussion

We designed an *in-vitro* evolution experiment where cells are selected at successively increasing sublethal levels of norfloxacin. Using such an experimental paradigm, *E*. *coli* evolves in the laboratory in a mode that may mimic one of the pathways by which bacteria become resistant to clinically significant levels. Evolution of resistance is gradual and due to accumulation of multiple mutations, four of them in the well-known targets of quinolones, namely DNA gyrase (*gyrA*) and DNA topoisomerase (*parE*) and others affecting expression of cell wall porins and AcrB. This experimental setup is different from the reality in the clinics where patients are given high doses of antibiotics, so the question may be raised as whether it is relevant. The finding that the mutations identified here are identical to many of those identified in the clinics provides a strong validation of our experimental paradigm. Moreover, selection of resistant bacteria does not necessarily start in the clinics. It has been shown to occur at extremely low antibiotic concentrations [34], concentrations present in many environments because of the widespread uncontrolled use of antibiotics. In addition, subinhibitory concentrations of antibiotics have been shown to modulate levels of a large number of transcripts [35].

**Fig 5:**
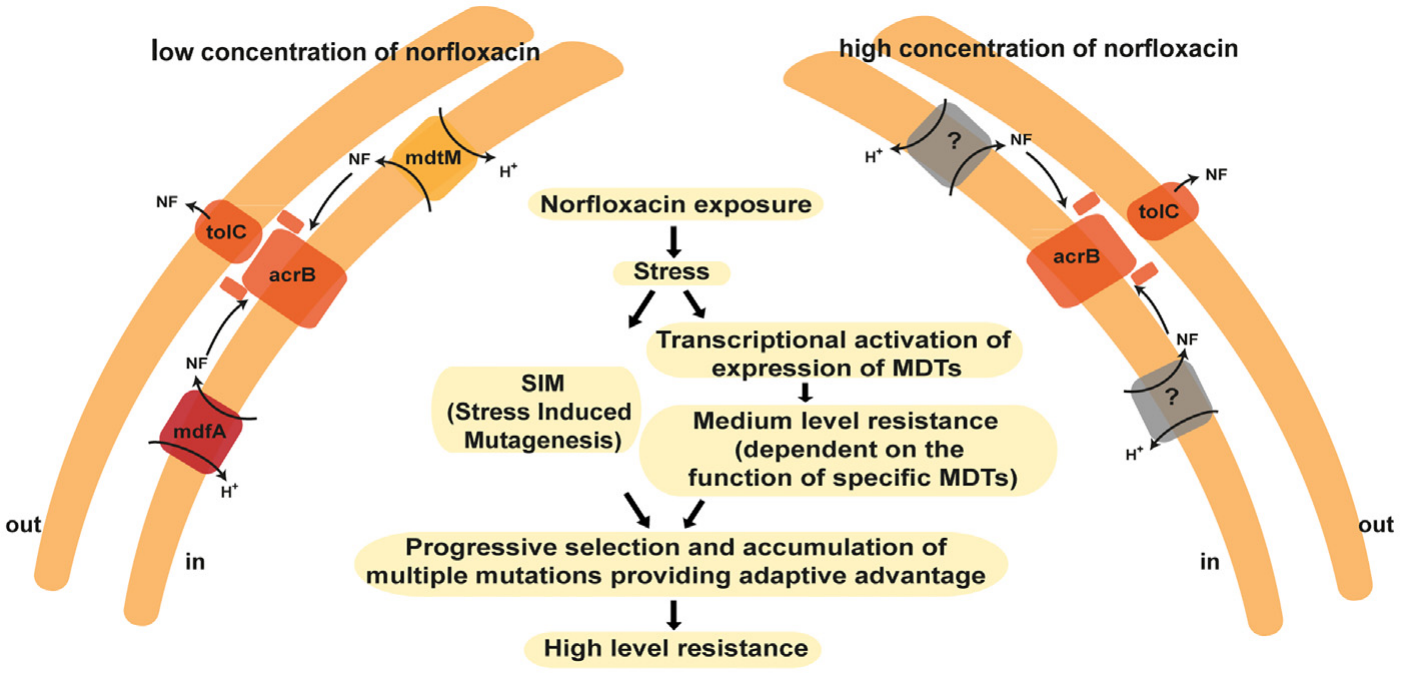
Sequence of events leading to high-level resistance. In this sequence, the role of the MDTs is to allow fixation of mutations that otherwise would be lost in the next increase in concentration.

A crucial conclusion of these experiments is that the function of single component MDTs, the suppliers, is essential for the process of acquisition. A massive majority of the work regarding the role of MDTs in AMR in Gram-negatives has focused on the AcrAB-TolC complex because of the minor role previously reported for individual single-component transporters. However, the use of multiple nil mutations of single-component transporters with overlapping specificities has demonstrated that they play a fundamental role in supplying the drugs to the AcrAB-TolC complex [18]. Remarkably, as shown here, the selected transporters do not play any assessable role in the maintenance of the high-level resistance. It is possible that after the multiple mutations have accumulated there is no need to remove the drug. However, a more likely explanation is that MdtM and MdfA function only at low concentrations of norfloxacin (“high affinity”) while other low-affinity transporters, not yet identified, remove norfloxacin from the cytoplasm at the very high concentrations that the evolved strain can withstand (Fig 5). The latter possibility seems more appealing because the function of AcrAB-TolC, that requires collaboration with suppliers, is significant for maintenance of a fraction of the resistance. The search for the low-affinity transporter(s) can now be carried on using the strains developed here.

The undisputable central role of the AcrAB-TolC transporter in the susceptibility of *E*. *coli* cells to multiple antibiotics has been documented in many ways. In many instances it was shown that expression of AcrB, and at times other MDTs, is increased in resistant strains leading to an associated increased efflux of antibiotics from the bacterial cell [9]. Recently, for the first time it was also shown that a mutation in the gene coding for AcrB modifies its substrate specificity and confers resistance to ciprofloxacin [36]. In our work we confirm previously reported findings that document the overexpression of AcrB when the wild type strain is exposed to the antibiotic and we also show that the increased activity of the AcrAB-TolC complex contributes to the high-level resistance. However, in a nil AcrB strain, high-level resistance is achieved to levels only slightly lower than those achieved by the wild type strain and we show that this is most likely due to the overexpression of other TolC-dependent transporters, mainly AcrF. This was the case also when chloramphenicol was used as the selecting agent. Similar findings have been reported when in vitro selection experiments with *acrAB* knockout strains yielded resistant mutants after a single step. Enhanced efflux in these mutants was due to increased expression of AcrEF [37] and this has been suggested to be a general response of TolC dependent transporters including also AcrD as shown here [38]. Similarly, in *Salmonella typhimurium* it was shown that the expression of all RND efflux pump genes was increased when single or multiple *acr* genes were inactivated, suggesting a feedback mechanism that activates the transcription of homologous efflux pump genes [39]. Our conclusion in this respect is in apparent contradiction with a previous report that claimed that topoisomerase mutations are ineffective in an AcrAB nil background [40]. Our strains and experimental paradigms were very different but, most importantly, our selection lasted for a longer time and allowed for adaptive steps in the nil AcrB strain that became apparent only after 14 days (Fig 1). In this context, it would be advisable to test whether in cells where AcrB is rendered inactive by point mutations or by specific inhibitors there is, under selective conditions, a similar compensation mechanism. We anticipate that these findings and further research of this topic will influence the strategy for the search of inhibitors of AcrB as adjuvants to existing antibiotics. We propose that inhibitors of one or two suppliers, specific for a group of antibiotic, may prove to be also efficient as adjuvants since, at least in our experimental setup, we have observed a complete dependence on the suppliers for the acquisition process. The likelihood of finding a common inhibitor for two or even three suppliers is not so unconceivable since they overlap in their specificity.

Our work here highlights again the fact that assessment of the role of transporters in AMR requires a clear distinction between three different resistant phases: naïve, medium-level and high-level, clinically relevant, AMR. While the evidence that supports the role of AcrAB-TolC complex in the naïve state and medium-level resistance is unquestionable, here we demonstrate experimentally an incremental role in high-level resistance (see also [40]). However, we must caution that in nil AcrB mutants a compensating increase in other transporters is detected so that acquisition of resistance is not affected. Moreover, despite the clear contribution of AcrAB-TolC function to the high-level resistance, the nil derivative of the evolved strain still displays a high resistance that increases rapidly upon further exposure to the antibiotic.

Our present interpretation of the process of acquisition of AMR based on the experiments described here is summarized in Fig 5. The stress induced by exposure of cells to sublethal concentrations of norfloxacin has a dual effect. In parallel to a transcriptional activation of expression of MDTs, stress induced mutagenesis increases the mutability in the population. Only a mutation that confers advantage can be fixed when the challenge is increased by further rise of the concentration. The likelihood of such a mutation to be fixed is amplified when the MDTs actively remove some of the offending compound and thereby allow the cells bearing that mutation to rapidly multiply. Importantly, in this work we identified two of the MDTs capable of fulfilling this function when exposed to norfloxacin, we provided strong support for the concept that suppliers and AcrAB-TolC type of transporters must work in a concerted mode and we showed that the function of the key transporter AcrAB-TolC can be partially backed up by other TolC-dependent ones. In the absence of the appropriate MDTs the advantage edge is lost in the next step of the evolutionary process.

## Conclusions and Questions for the Future

The role of MDTs other than AcrAB-TolC in Gram negatives has been frequently estimated as marginal. However, it is becoming evident that the function of the AcrAB-TolC complex requires coordination with the function of single polypeptide MDTs, the suppliers [18, 41, 42]. The unquestionable central role of AcrAB-TolC is due to the fact that it functions in a funnel-type manner in the last of a 2-step process where it removes noxious substances from the periplasm to the cell exterior after a number of transporters remove them from the cytoplasm. We anticipate that for each family of antibiotics there are at least one or more, suppliers responsible for the cooperation with the AcrAB-TolC complex. A fascinating consequence of this finding opens up the question as what are the specifics of this interaction and whether efficient capturing of hydrophobic substrates from the periplasm or from the external leaflet of the membrane necessitates direct, physical interaction of the transporters. Are these transient interactions or preexisting ones and in the former case, are they induced by the nature of the external noxious stimulus or are they random?

## Supporting Information

S1 Fig Medium level cross-resistance to antibiotics other than quinolones. IC50 values for erythromycin and chloramphenicol were determined for the naïve and evolved states and the nil triple mutant of the WT-EV18.
(PDF)

S2 Fig Evolution of high-level resistance to chloramphenicol. Cells were grown overnight in the presence of 0.1 μM chloramphenicol and cultures that reached at least A600 = 1 were further diluted to LB-KPi containing twice the chloramphenicol concentration. The process was continued with a daily two-fold increase in the concentration for 24 days. Cells that did not grow at the higher concentrations were kept at the permissive ones and retested again after 24 hours. Inset: IC50 values were determined as described in Methods.
(PDF)

S1 Table Primers used for the determination of transcript levels. (PDF)
