# Supplementary figures and images for: A Transporter Interactome Is Essential for the Acquisition of Antimicrobial Resistance to Antibiotics
Source: PLoS One. 2016 Apr 6;11(4):e0152917. doi: 10.1371/journal.pone.0152917 (PMC4822809; doi:10.1371/journal.pone.0152917)

**S1 Fig. Medium level cross-resistance to antibiotics other than quinolones.**

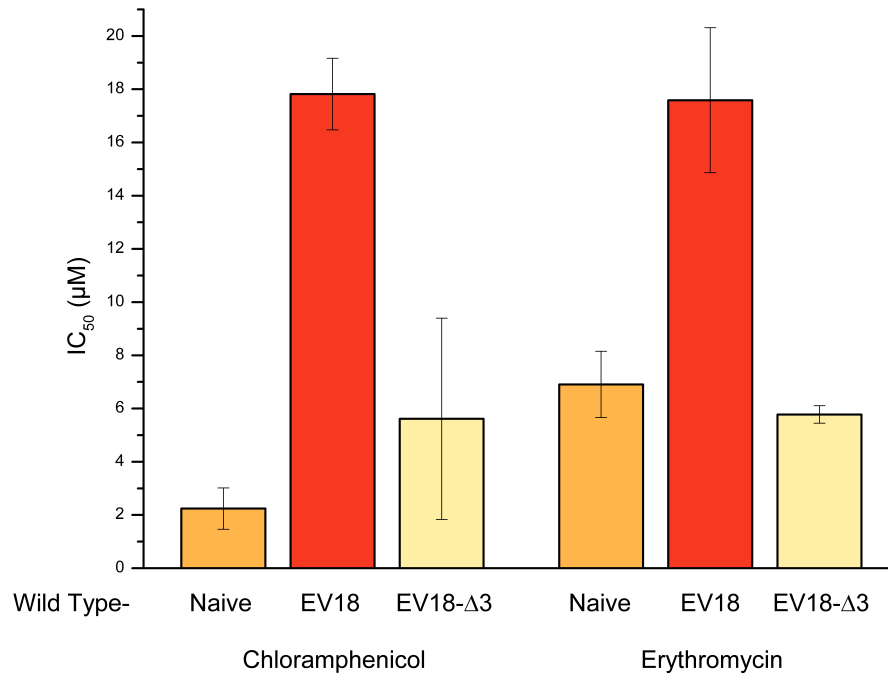

Supplement: S1 Fig — IC50 values for erythromycin and chloramphenicol were determined for the naïve and evolved states and the nil triple mutant of the WT-EV18. (PDF) [file pone.0152917.s001.pdf]

**S2 Fig. Evolution of high-level resistance to chloramphenicol.**

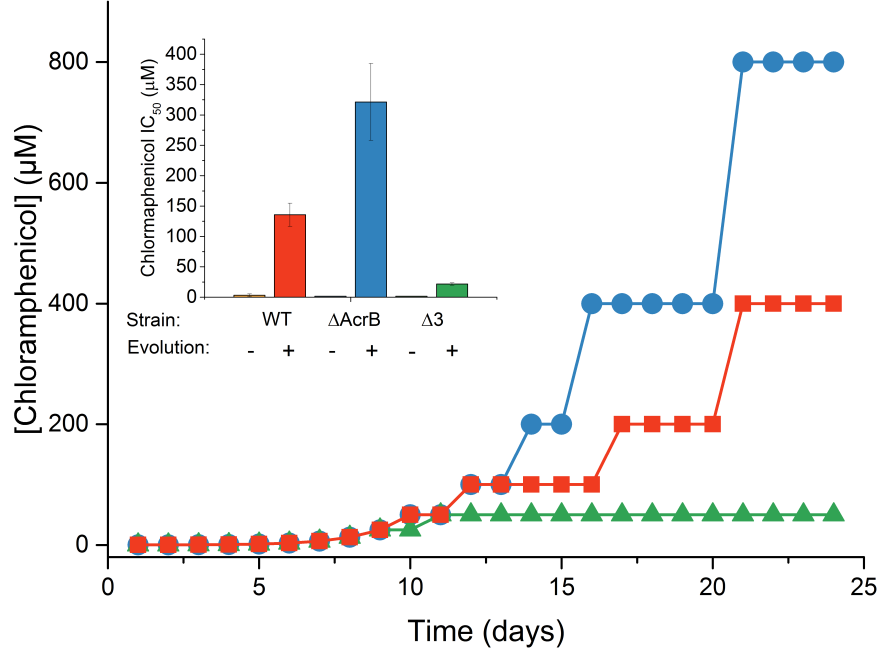

Supplement: S2 Fig — Cells were grown overnight in the presence of 0.1 μM chloramphenicol and cultures that reached at least A600 = 1 were further diluted to LB-KPi containing twice the chloramphenicol concentration. The process was continued with a daily two-fold increase in the concentration for 24 days. Cells that did not grow at the higher concentrations were kept at the permissive ones and retested again after 24 hours. Inset: IC50 values were determined as described in Methods. (PDF) [file pone.0152917.s002.pdf]
