# Supplementary material for: A Transporter Interactome Is Essential for the Acquisition of Antimicrobial Resistance to Antibiotics
Source: PLoS One. 2016 Apr 6;11(4):e0152917. doi: 10.1371/journal.pone.0152917 (PMC4822809; doi:10.1371/journal.pone.0152917)
Supplement: S1 Table — (PDF) [file pone.0152917.s003.pdf]

S1 Table Primers used for the determination of transcript levels.

Primer	Sequence
F-qRT-acrB	GAAGAGCACGCACCACTACAC
R-qRT-acrB	GCAGACGCACGAACAGATAGG
F-qRT-acrF	GCGCGTGATGATGGAGGATA
R-qRT-acrF	AAATACCGCTGACAGCACCA
F-qRT-mdtF	CTGATGGTAGCGGCGTTTAT
R-qRT-mdtF	GGCATACTCGGAACCAAAGA
F-qRT-macB	GGCTGGAAGACCGTACAGAG
R-qRT-macB	GTTGGTTCATCGGCAAGAAT
F-qRT-gapA	GGACGAAGTTGGTGTTGAC
R-qRT-gapA	TTCTGAGTAGCGGTAGTAGC
